# Exploring the stigma of mental illness in life worlds of different economic pressure: a maximal contrast analysis

**DOI:** 10.3389/fpubh.2026.1677166

**Published:** 2026-05-18

**Authors:** Sven Speerforck, Claudia Helmert, Anya Leonhard, Matthias C. Angermeyer, Bruce G. Link, Ingo Matuschek, Georg Schomerus

**Affiliations:** 1 Department of Psychiatry and Psychotherapy, Medical Center, University of Leipzig, Leipzig, Germany; 2 Center for Public Mental Health, Gösing am Wagram, Austria; 3 Department of Sociology, University of California, Riverside, Riverside, CA, United States; 4 Department of Sociology, University of Applied Labour Studies (HdBA), Schwerin, Germany

**Keywords:** economization, life worlds, mental health, mental illness, social structure, stigma

## Abstract

**Introduction:**

Little is known about how differences in economic pressure in life worlds shape understandings of mental illness and stigmatization toward people with mental illness.

**Methods:**

We conducted an explorative study using five focus groups with three to five participants selected through purposive theoretical sampling. Each focus group represented a different life world (operationalized through shared occupational and sociopolitical life goals). Using the method of maximum contrasts, we conducted a qualitative content analysis of 2 focus groups (group 1: *n* = 4, mean age: 37.7y, volunteer fire fighters; group 2: *n* = 4, mean age: 41.3y, self-employees working in the service and media sector).

**Results:**

Different life worlds corresponded with different manifestations of stigma and interactions with people with mental illness. Self-employed entrepreneurs rationalized their social behavior using a cost–benefit-analysis that considers possible costs regarding performance and self-optimization. In contrast, volunteer fire fighters described trying to integrate people with mental illness in a familial way.

**Conclusion:**

Life worlds not only interrelate with stigmatizing attitudes but shape the process of stigmatization itself. These first results can be used to design modern questionnaires to examine stigmatization and support targeted intervention and prevention strategies.

## 1 Introduction

Stigmatization is a social process (1) that shapes societal attitudes towards mental illness. However, stigma itself is also shaped by the social context in which it occurs. Goffman described “stigma-management [as] a general feature of society” (2). Central to this social function of stigma is power (3). Power is exerted through stigma in order to keep people in, down or away (4): Stigma ascribes less power and fewer resources to people (to keep people down), perpetuates conformity to social norms (to keep people in) and reveals ways to avoid (assumed) diseases or danger (to keep people away) (4). Thus, stigma contributes to and reflects social inequality. The interrelation of stigma and social context is also reflected in the “What matters most” approach by Yang et al. (5), which focuses on aspects of stigma that block individuals from meaningfully participating in routines that are essential for establishing “personhood” within a specific cultural context (e.g. “moral experience”) (6). Engaging with work—both as a way to make money but also as an occupation that takes up a person’s time—is described as one of the most important experiences within this context. Although Yang and colleagues focus on cross-cultural comparisons, they also acknowledge that microsocial contexts shape experiences of stigma (5, 7).

Building on this, we approach life worlds, also known as “micro-realities” or “micro-worlds” as a microsocial context (8). Life worlds are defined as groups that show a certain stability over time and whose members share common experiences, values, attitudes, and who pursue similar goals. Life worlds can be used to operationalize social inequalities, as they constitute shared interaction spaces and everyday environments (9). Life worlds, as small-scale societies, are likely shaped by and shaped through their inhabitants’ attitudes and behaviors toward individuals with mental illness. Returning to Yang et al. (5), they describe work engagement—making money, spending time—as one of the most important aspects of their “What Matters Most” theory. In this paper, we explore how life worlds, specifically pertaining to work engagement and economic status, may affect people’s interactions with mental illness. As work engagement is a broad field, we define it as a latent, overarching concept that shapes life worlds, and highlight economic pressure as one specific and empirically traceable facet. Work engagement is also a relevant concept for life worlds due to the economization of the social sphere, which has also been associated with mental illness (10, 11). The economization of the social sphere has been shown to vary between different social groups and has been associated with discriminatory attitudes, further highlighting the relevancy of work engagement for understanding stigma. One reason for linking the economization of the social sphere to life worlds can be found in research by Groß et al. (12). They report that people with different social statuses differ in their economization of the social sphere, which in turns is associated with discriminatory attitudes.

In our study, we combined different conceptual as well as empirical approaches to understand the stigmatization toward people with mental illness in modern times. Previous qualitative studies on mental illness stigma have primarily focused on the perspectives of affected individuals or examined stigma within clinical or institutional settings (13). Studies addressing the role of social context have largely remained at the level of broad cultural or national comparisons (5, 14). Systematic qualitative explorations of how distinct microsocial milieus within the same society differ in their engagement with mental illness stigma is missing. Recent work by Wu et al. (15) has begun to examine stigma in organizational settings from a sociolinguistic perspective. Pybus et al. (16), on the other hand, investigated how macro-economic conditions relate to stigma across European countries. Our study complements and extends these efforts by shifting the analytical focus to life worlds at the meso-level having an influence on microsocial contexts and shape stigma processes from within.

We aim to address several research gaps in the existing literature: First, while population-based attitude research has extensively documented stigmatizing attitudes at the macro level (17), there is a notable lack of research on small-scale social contexts and group processes, such as life worlds. Life worlds are particularly relevant in modern times, in which social structure becomes more complex and is diverging (2). We aim to explore how these micro-social contexts are associated with stigmatization toward people with mental illness (17, 18). Second, we aim to explore how stigma’s social functions as a form of power and as collective representations manifest within these contexts (4, 19). Further, we will explore how and to what extent the economization of the social sphere affects different life worlds, as well as whether this relates to the stigmatization toward people with mental illness.

Communication is essential for creating and perpetuating stigma as well as for disclosing and coping with existing stigma (20). Differences in how people in different life worlds talk about mental illness and stigma have not been described sufficiently (21, 22). Thus, we also examine how people communicate about and engage with mental illness and stigmatization in different life worlds. To our knowledge, our case study is the first to examine how life worlds shape the stigmatization toward people with mental illness.

## 2 Research question

From this theoretical background, we developed the following research questions:

Are there different strategies in engaging with mental illness in different life worlds?

Are different life worlds reflected in different manifestations and functions of stigma toward people with mental illness and disability in general?

## 3 Materials and methods

### 3.1 Study design

To address our research questions, we used an exploratory qualitative study design. Through focus group discussions, we aimed to evaluate how people inhabiting different life worlds talk about people with mental illness. Focus groups are therefore a useful tool to study excerpts from everyday life, each focus group represents a different life world. In the following section, we describe our approach according to the Consolidated Criteria for Reporting Qualitative Research (COREQ) for interviews and focus groups (23).

### 3.2 Participant selection

The aim of our study was a targeted selection through theoretical sampling (24) of people with shared life worlds. The guiding principle for data collection was participants’ orientation toward life worlds (8, 9). Life worlds are likely a matter of locally anchored and permanent associations between subjects, which people join voluntarily and in which values are, to a greater or lesser extent, shared with other members. As mentioned in the introduction section, our focus is on moral and economic aspects, based on research by Yang et al. (5, 6). These researchers highlight that these aspects matter most in the stigmatization toward people with mental illness. To evaluate life worlds with shared occupational and sociopolitical goals, including latent economic pressure, we operationalized different life worlds through individual focus groups As studies on left-leaning, more affluent life worlds and their role for creating health inequalities are scarce (25), we began our recruitment process there. However, we had no concrete exclusion or inclusion criteria, except that the socio-economic backgrounds of participants should be different. This is consistent with our exploratory approach.

Until we achieve theoretical saturation, we recruited participants from our broader social environments. Theoretical saturation was assumed by consensus of researchers (SS, IM, MCA, GS, and CH) during discussions following the focus groups (find details below, in section 3.4). Our indication for theoretical saturation was that no new findings regarding how people negotiate stigma and behavior toward people with mental illness emerged, no additional codes and topics came up with the fourth and last focus groups. The central theme that kept coming up was study participants’ perception of restrictions on individual freedom which accounts for more stigmatizing behavior. We asked people if they would be willing to participate in a group discussion about the topic of “people with disabilities and emotional stress” (26). The topic was described in general terms to reduce selection bias.

The following focus groups were evaluated: employees of a psychiatric clinic (*n* = 3; identification in the following text, interview group/persons (ident): A/1–3), a local branch of a political party and acquaintances (*n* = 4; ident: B/1–4), a study service organization (*n* = 5; ident: C/1–5), representatives of a volunteer fire brigade (*n* = 4; ident: D/1–4) and founders, owners and self-employees of start-ups in the service and media sector (*n* = 4; ident: E/1–4). The latter two groups (D/1–4 und E/1–4) were selected for maximum contrast analysis, because they differed the most in their moral concepts and how they talk about people with mental illness. No participants dropped out. No people invited to the focus groups refused participation.

### 3.3 Procedure

Group discussion procedure mainly followed Bohnsack (27). Discussions were conducted by CH (female PhD student) and started with an initial prompt:

In everyday life, within family, in our leisure time and at work, we meet a wide variety of people. Among others, we see people with physical disabilities, but we also meet people with emotional distress. Regardless of how important these people may be to us, it is often necessary to engage with them. (For example, sometimes, we avoid people because we are under time pressure to get to an important appointment).

Do you have any experience in this regard? Did you meet people with disabilities and/or stress in your family, leisure time or at work? If so, how?

Afterwards, the discussion continued prompted by two vignettes. The first describes symptoms experienced by someone with depression, while the second describes symptoms experienced by someone with schizophrenia. These vignettes served as minimally interfering impulses and were adapted from previous studies in stigma research [for vignette validation, please see (28)]. As the vignettes are realistic and describe symptoms, it is not necessary to recognize or identify the illness in order to discuss them. Some of the individuals in the groups picked out clues from the vignettes that they could relate to their own experiences. Overall, our discussion guide included the initial prompt, the two vignettes, and specific questions. This hybrid constellation of methods was intended to allow the greatest possible freedom in response behavior within a set thematic framework.

From the perspective of the study, the selection made within the framework of the aforementioned iterative sampling procedure and the insights gained from the survey were appropriate. The focus group discussions lasted 1.5 and 4 h. Discussions took place in person at the focus groups’ work surroundings, except for the discussion with the study service organization, which was conducted via video conference. No interviews were repeated. Field notes were taken during discussion. Conversations were audio recorded and transcribed by a writing agency (we have no information about the number of data coders). Quotations from the German-language discussions and transcripts were translated into English by a native English-speaking colleagues (AL) (German originals are listed in the Supplementary Table S1). Transcripts were not returned to participants; no comments or corrections were made possible in order to reduce the influence of social desirability which is of particular interest in stigma research, because stigmatization “might pose a threat to personal beliefs of open-mindedness” (29) p. 186.

### 3.4 Analysis of maximal contrasting groups

The transcripts of each focus group were discussed by the researchers (SS, IM, MCA, GS, and CH) and divided into sequences of different lengths. This initial interpretation step helped to develop the coding manual. The coding manual included deductive codes (based on the interview guide), which were complemented by inductive codes (derived from data). The subsequent coding was internally validated through comparison with independent coding by another person (AL, female MD student experienced in qualitative research). Non-conformities were systematically discussed and consequences for the category system and coding were documented. The analysis was performed with the two researchers’ (AL, CH) reconciled coding. This corresponds to the method of qualitative content analysis specified by Kuckartz and Rädiker (30), which oscillates between interpretative and rational analyses of interview data [on such mixed types, please see (31)]. Additionally, we evaluated how participants verbally expressed their shared everyday knowledge during group discussion (e.g., through rhetorical means like metaphors, similes). Participants had no possibility to intervene with results or to provide feedback on findings, which reduces the influence of social desirability on answers. We chose a maximum contrast analysis, comparing the two focus groups that differed most in terms of their social context and how they talked about people with mental illness. Our aim is to demonstrate the variety of our findings between the two extremes of this spectrum, the two maximal contrasting focus groups (32)—the aspects that differ most significantly in people’s lived experiences and their accounts of interacting with individuals with mental illness.

### 3.5 Software

The data was read into MaxQDA (33), coded and analyzed, both for individual cases and across cases, always in the context of the complete focus group discussions.

### 3.6 Ethical statement

All research conducted in this paper received ethical approval from the Ethics Committee of the Medical Faculty of Leipzig University (112/22-ek).

## 4 Results

### 4.1 Sample

Demographic data for all five focus groups is shown in Table 1. Maximum contrast of two selected groups can be seen in differences between professional qualifications (D/1–4: Vocational training (apprenticeship) completed and E/1–4: University or college degree) and mean income (overall mean income per participants’ household for each month: D/1–4: 3079€; E/1–4: 28333€) when compared to the other groups. The number and gender of participants in the two maximally contrasting focus groups are equal (*n* = 4, all of them men). Since the topic of work engagement, a latent concept underlying our conception of life worlds, is very broad, we focused on economic pressure and the economization of the social sphere to better understand different focus groups. These aspects emerged during analytic interpretation and were observed during the group discussions. In the following, we link these differences to how different life worlds engage with mental illness.

**Table 1:** Sociodemographic data (sex, age, educational and professional qualification and status, income) and an exemplary quotation describing 5 focus groups and life worlds, *n* = 21.

Group		*n*=		Age (mean)	Highest school qualification (median)	Highest professional qualification (median)	Professional status (median)	Income (netto, per study participants’ household) (mean, €)	Exemplary characteristic quotations as focus groups’ self-description regarding behavior towards people with mental illness and disability
		f	m						
A/1–3	Employees of a psychiatric clinic	2	1	35.3y	Abitur/general or subject-specific higher education qualification (Gymnasium or EOS, also EOS with apprenticeship)	University or college degree	Employee with independent performance in a responsible position or with responsibility for personnel (e.g., researcher, head of department or foreman in an employment)	2250.00 (missings: *n* = 0)	*keeping your distance for self-protection* (A1, A2, section 91, section 92)
B/1–4	People of a local branch of a political party and acquaintances	2	2	31.5y	Abitur/general or subject-specific higher education qualification (Gymnasium or EOS, also EOS with apprenticeship)	University or college degree	Employee with independent performance in a responsible position or with responsibility for personnel (e.g., researcher, head of department or foreman in an employment)	2175.00 (missings: *n* = 0)	Mod: *But when you say you would ask which doctor they go to, with what intention are you doing that? Or what kind of answer do you expect?* B2: *Well, I think I would invent a story around it so as not to be offensive [übergriffig]. (…)* B1: *Cool move.* (Moderator, B2, B1, section 276–280)
C/1–5	Employees of a study service organization	5	0	54.0y	Abitur/general or subject-specific higher education qualification (Gymnasium or EOS, also EOS with apprenticeship)	University or college degree	Employee with independent performance in a responsible position or with responsibility for personnel (e.g., researcher, head of department or foreman in an employment)	5500.00 (missings: *n* = 3)	*There needs to be a paradigm shift so that it is the healthy person’s responsibility to ensure that these people with disabilities feel comfortable, and not the other way around.* (C2, section 85)
D/1–4	Representatives of a volunteer fire department	0	4	37.7y	Secondary school leaving certificate (“Mittlere Reife”) or equivalent qualification	Vocational training (apprenticeship) completed	Employee with independent performance in a responsible position or with responsibility for personnel (e.g., researcher, head of department or foreman in an employment)	3078.75 (missings: *n* = 0)	*It’s like one big family here, and that’s how it should be everywhere.* (D1, section 56)
E/1–4	Self-employed people working in the service and media sector	0	4	41.3y	Abitur/general or subject-specific higher education qualification (Gymnasium or EOS, also EOS with apprenticeship)	University or college degree	Self-employed and have/had 1 to 4 employees	28333.33 (missings: *n* = 1)	E4*: What is the opposite of constraints [Einschränkung]? Inspiration, Promotion?* E2*: performance.* (laughs) (E4, E2, section 118–119)

### 4.2 Coding tree

As mentioned above, the transcripts of each focus group were discussed by the researchers (SS, IM, MCA, GS, CH), divided into sequences of different lengths. This initial interpretation step helped to develop the coding manual and the coding tree. The coding was validated through comparison with independent coding by another person (AL, female MD-student experienced in qualitative research). This leads to a Cohens Kappa of 0.21 (if the number of codes per segment is unequal, or if a single code is being evaluated and with at least 50% code overlap) over all five focus groups, according to Landis and Koch (34) indicating a fair agreement between two coders. We discussed differences, clarified open questions and evaluated uncertainties to reach a consensus, which lead to some re-codings. Differences were due to different lengths of coded segments, segments were assigned to either superordinate or subordinate codes within the coding tree, or they were assigned to more than one code.

We generated 31 codes. 19 codes were identified in advance (deductive based on the interview topics), to denote answers that we wanted to compare between different focus groups. 12 codes were derived from collected data (inductive), because we did not expect these focus points in discussions. These inductive codes were developed during discussion with researchers (SS, IM, MCA, GS, and CH) and became part of the coding manual and coding tree. Top categories were experiences with people with visible and non-visible disabilities, perceptions or concepts of depression and experiences in engaging with people with depression, perceptions and concepts of schizophrenia and experiences in dealing with people with schizophrenia, metaphors the participants used to describe (their behavior towards people with) mental illness, and conclusions (individual and perceived societal moral standards, differences and similarities of depression and schizophrenia, concepts of freedom regarding people with mental illness). All codes and subcodes and the number of coded segments can be found in Supplementary Table S2. For the results we condensed these codes, the comprehensive coding tree, and patterns in the dataset according to the points which are mentioned in the headings below. The selected quotes are examples, which the research team considered as representative for the sound and language of the focus groups and to answer to the research questions.

### 4.3 Life world specific strategies to engage with mental illness and disability

#### 4.3.1 Life world of volunteer fire fighters

The life world of voluntary fire fighters is characterized by solidarity and responsibility: “We are a community. We want to go into the fire together. We want to come out together” (D/1, section 61). Group discussion revealed that they find it easy and rewarding to include people with disabilities and/or mental illness in their community. Their awareness is potentially influenced by own mental and physical crises they experience during their tasks as fire fighters (extinguishing fires, being dispatched to accidents and suicides). We suggest that their conceptualization of mental illness is influenced by participants’ experiences of being confronted with demanding situations, which is accompanied by empathy toward colleagues who may experience similar circumstances:

I would say the advantage you usually have—as I always say—is a good network in a “blue light family”. We had a serious house fire here in [town, Erzgebirge] a few years ago, where our local fire brigade also rescued a dead body. And one of our comrades struggled with this afterwards. And a colleague, who worked in another fire brigade, called me and said: “I know you are at the Christmas party. Please, talk to him again; we are concerned about his condition. So, if you have the chance, to talk to him or offer to talk to him. That also includes having a beer in the evening together. (D/4, section 110).

Regarding the label of disability in general, the group of volunteer fire fighters referred to categorical understanding of disability as opposed to a more continuum-like concept of disability, and sometimes used direct and potentially offensive language:

Yes, people with disabilities are the most honest and grateful people to me. And sometimes you can lower your expectations quite a bit. (D/4, section 30).

Regarding recovery, participants spoke about being trained and supervised by crisis intervention services (D/4, section 144). This might additionally support a sensitive approach towards people in need and with symptoms of mental illness. The volunteer fire fighters expressed wanting to help and being aware of people in need in a holistic way and saw the community as responsible for facilitating recovery. Their reports imply an adaptability to varying wishes and requirements of people with disabilities such as mental illnesses. One study participant emphasized:

Well, the most important thing that often does not happen the way you want it to is actually to analyze the root cause [of the problem]. We used to say that when my fellow human being crosses my path and has changed, then I feel that you usually treat the symptoms. Like this: If you are depressed, you have to take mood enhancers. Let us see, some kind of medication or something. But it’s actually important to have a conversation with people. (D/4, section 105).

#### 4.3.2 Life world of self-employees

In contrast to this sense of belonging to a community, the life world of self-employees in the media sector is characterized by individuality, performance pressure, and competitiveness. Thinking about their concept of cause of mental illness, we highlight the talk of E/1 and E/3 (section 496–497) who state:

E1: On the one hand, I believe our lifestyle causes more and more people to get sick. You know, it’s like we all live in a world where you can afford everything and where everyone can independently pursue their dream. But on the other hand, I think that a lot of people are unsatisfied with the sense of “I’m not in a community anymore and…”

E3: But the question is: is that a clinical diagnosis?

Symptoms of mental illness and disability are seen to be on a continuum:

Everyone has a little bit of something. But the question really is, when you say “limitation”: Is that something that you really, actually notice, where you say: Wow, this person cannot function at 100 per cent capacity? (E/3, section 48).

Considering recovery, this focus group talked about giving a person with mental illness the time to individually deal with his or her problem, highlighting the importance of medication and healthcare institutions for people with mental illness. Further, the self-employees in the media sector prioritized not overemphasizing the illness to avoid encouraging people with mental illness to adopt the “sick role.” They negotiate how they deal with people with mental illness and perform cost–benefit-analyses, whereas the costs can be described by study participants own resources (time, maintaining their ability to work) and benefits constitutes assumed benefits for the person in need: “How much effort do you want to put into entering (t)his world and saying: Do I want to change something or not? The question is: Can you change anything at all?” (E/3, section 455). Nevertheless, their negotiations remain hypothetical, indicating more interest in talking about than helping people, as E/2 summarizes: “it simply remains a mental exercise as long as you do not act accordingly.”

Our analyses reveal that these two exemplarily life worlds differ in engaging with mental illness and disability. Volunteer fire fighters were more community oriented and highlighted the importance of including people with mental illness. Participants from the media sector weighed up individual costs and benefits in engaging with and including people with mental illness and seemed to be more performance-oriented.

#### 4.3.3 Life worlds reflected in stigmatization toward people with mental illness

To examine how different life worlds are reflected in different manifestation of stigma, we summarize our results on experiences and expectations toward people with mental illness, individual and societal moral concepts, and images of mental illness and behavior toward people with mental illness. As stigma is a way of dealing with deviations from norms (3), we found that the norm of individual freedom varied between life worlds. In contrast to the volunteer fire fighters, the focus group of self-employees in the media sector were particularly concerned with the freedom of people with mental illness to deal with their problems or to recover on their own (E/2–4, section 324–328):

E2: Well, even with depression, it’s sometimes hardest to help those who are close to you >.

E4: Sure, because you … The shame, so to speak, is the greatest of all.

E2: The shame. But you also want to somehow. Somehow you do not want to do anything to them that they do not want, supposedly.

E3: You might not even see it at some point.

E2: Maybe you do not see it at all.

They considered if they support encouraging help-seeking or respect the freedom and individuality of the person affected. Study participants emphasized that a person with depression has (personal) boundaries they do not want to cross. This seems to be a reason for not helping people with mental illness, as the following excerpt illustrates (E/1–3, sections 343–349):

E3: Are you the one who takes care of him, or someone else? (short pause).

E1: Are we responsible now for taking care of him, can we manage it? But is the problem not also a little bit our society, that we always assume that everyone…

E3: Someone will deal with it, you mean?

E2: There’ll be something that organizes it.

E1: No that you say, well, privacy, and I need to… like when do I invade his privacy? Like in the example [vignette], just calling and saying „here, please get him to hospital, it’s just a blatant… well… um…

E2: Yeah.

E1: Then it’s always like: before I do something wrong, I might as well do nothing at all.

In line with their values of freedom and personal fulfilment, performance and work functionality drive self-employees’ behavior (referencing the neoliberal labor market, as we will discuss later). This may be disabled by (mental) illness and includes the most potentially relevant stereotypes in this life world:

I always have a problem with people who do not think logically, and when you are that ill, you just do not think logically anymore. (E/1, section 447).

In the first bit we already talked about not being able to rely on people anymore, which in a professional context has way more consequences. So that, what [E3] just said about his colleague that he took with him to the job, who did not deliver and then was out [of a job], it would be the same with me. Like, if I had someone who said: I need this and that. Or I tell him: I need this and that by the day after tomorrow. And if that does not happen two or three times then it’ll definitely have the consequence that I would not work with that person anymore. And if that was, for example, my wife, then that would not be the case because I’m there for her no matter what. Well, no-matter-what is also kind of in the air because at some point that’s over, too. (E/2, section 215).

In contrast to the participants in the focus group of self-employed people, the volunteer fire fighters were characterized by their self-conception as lifesavers and as a crucial part of the community. They do not have to compete in a (neoliberal) labor market, economization plays less of a role this context. They describe people with disabilities and mental illness as people they must find a way to get along with and acknowledge that it is important to be aware of their needs and thoughts. They look for niches in which people with disabilities can contribute to the community, to make them feel safe and not negligible (D/4, interviewer, D/1, sections 23–25):

D4: So you have to come up with something to keep them happy anyway, like saying: You’re now a passive member with these tasks or we’ll find opportunities in other areas of the fire service. There are I mean, we even have musical platoons. Maybe there’s a musical solution where you can integrate someone further and so on, and so on. I’d say there’s no limit to improvisation, which we are actually good at in the fire service. These are the most important points that went through my mind this evening when we when the enquiry came from you. And yes, there’s certainly no right or wrong way to put it. So it’s always an individual solution, where we cannot write a textbook, but perhaps rather, let us say, a catalogue of ideas of what someone somewhere has already tried and done to make this inclusion possible in this area.

Interviewer: And what have you already done or what are your suggestions? Also, if you have experience with it.

D1: Yes, well, as I said, we have thought about it, the other mates as well. So he will still be allowed to go with us [to the fire brigade operation], no matter when and how. Because he’s on fire and, as [name D4] says, he can do other duties [which are not at the scene]. I say, when we are called out for an operation, he is supposed to do the kitchen or something.

Therefore, life worlds reflect and shape the values that drive people’s behaviors. Further, life worlds influence the stigma toward people with mental illness when comparing volunteer fire fighters and self-employed people in the media sector.

## 5 Discussion

### 5.1 Familial and transactional relationships—life worlds matter for stigma research

The maximum contrast analysis revealed many disparities. The two focus groups have different approaches in how they talk about people with mental illness. While volunteer fire fighters focus on familial relationships and inclusiveness, the group of people working in the media sector refer to transactional relationships where work and functionality in society matters. Particularly the latter group shows that economic factors also determine social behavior. Consistent with Schütz and Luckmann’s (9) life world approach—which highlights the mutual shaping of people and their life worlds—our findings suggest that considering these latent contexts and circumstances helps to explain how stigma is manifested and how study participants engage with people with mental illness.

### 5.2 Economization of the social sphere permeates stigmatization processes

We identified interrelations between manifestations and functions of stigma toward people with mental illness in the two exemplary life worlds. In these life worlds, we employed economic pressure as a latent concept for participant selection. The volunteer fire fighters who participated promoted a community oriented and inclusive approach towards people with mental illness. In contrast, self-employees in the media sector discussed if and how to help people with mental illness, especially in the context of work performance. These findings are consistent with ongoing research on the discursive production of mental health stigma within organizational practices (15) and, on a broader level, on current social demands, such as the effect of a perceived meritocracy on stigmatization of mental illness (10).

To better understand the differences in life worlds, we suggest using the extent of economization to the social sphere as an interpretative lens. Compared to the group of self-employees, economic considerations played a lesser role for volunteer fire fighters from a rural region. Even though volunteer fire fighters have less income than self-employees, they do not stigmatize people with mental illness more, in contrast to results by Pybus et al. (16), which found that people with low SES are more stigmatizing. Our detailed qualitative approach helped understand how volunteer fire fighters conceptualized mental illness as a categorical, individual disability and considered various methods of integrating people with their needs into the community (“at the safe edge of the community”, D/4, section 30). Future work can discuss this within the framework of benevolent stigma (35) or benevolent othering (36). Nevertheless, firefighters’ experiences with crises, accidents and catastrophes may have shaped an awareness for certain symptoms of mental illness. Their overall goal when talking about people with disabilities seems to be inclusively working together as a community. They reported structuring their engagement through a willingness to learn about needs and competencies, and by maintaining the individuals’ capacity to act. Their main intention to help people with illness to establish and maintain community cohesion, which reflects the goals and approaches of the volunteer fire department as an essential community service.

In contrast, the self-employed study participants assessed the costs and benefits of their behavior toward people with mental illness. Economic pressure was not only an interpretative lens, but also an emerging theme that drives individuals’ behavior and their inclination to self-optimize. This may be because they work independently and prioritize competency (e.g., obtaining assignments, asserting themselves in a market of competent self-employed individuals) and their potential to compete with others. Our analyses are consistent with research by DeLuca et al. (37) and Papadopoulos et al. (38), who stated that individualism and competitiveness are associated with stigma toward people with mental illness. Further, the self-employees were driven by a “performance-imperative” (39): they rationalize and justify their investments, such as helping people with mental illness, and negotiate strategies for exclusive interactions. Their orientation is driven by self-economization and -optimization within the context of work. As “work-force-entrepreneurs,” Voss (40, 41) conceptualized these characteristics and describes the consequences of subjectification and economization on work in modern societies. Bröckling (42, 43) explored this concept and Zorn further developed it (44): rationalization and economization affect not only the workplace, but also almost all areas of life (39), including social behavior. Similarly, our results show that social pressures to compete and to self-optimize permeate social spheres.

Consistent with results from Yang et al. (5), we found that (voluntary) work and functionality matter most in stigma processes toward people with mental illness. This is why we employed eonomic pressure as a sampling strategy. Furthermore, it emerged as a key theme during the focus group discussions and provided a valuable interptetative lens through which to understand how it shapes the stigmatization process. In particular, the economization of the social sphere shapes the stigma process, especially for people in the media sector (45), as our research suggest. This is according to the modified labeling theory (46), which highlights that stigma and mental illness interrelate with the social environment, which in turn implies variations in stigmatization between life worlds. The close interrelation of life worlds and stigma makes targeted, life world specific actions against stigmatization toward people with mental illness particularly challenging, but necessary.

### 5.3 Stigma power and rhetorical modernization

Since qualitative research is based on the verbal conceptualization of stigma, we found noteworthy results concerning not only *what* people in different life worlds say about mental illness but also *how* people frame and articulate their helping behavior within their respective life worlds. Our goal was to address the research gap mentioned by Breshnan and Zhuang (21) and study how people communicate and negotiate stigmatizing thoughts and behavior.

Stigma and life worlds relate to values and conceptions of norms (as well as deviations and distinctions from these norms). This can be seen in the language people use. Self-employed participants in the media sector talk about depression as a brain disease and emphasize medication, as previously reported (47). Compared to the representatives of the volunteer fire departments, the focus group of self-employees was more subtle and cautious when discussing how to avoid helping people with disabilities. This may help to justify their behavior and avoid possible costs as deficits in their performance. They seem to be aware of modern discourses about mental illness and use appropriate wording when talking about their rejection of people with mental illnesses. This discrepancy between wording and behavior was conceptualized for instance by Wetterer (48) as rhetorical modernization. This may potentially reduce cognitive dissonance and maintain inequalities. The strategic conversation skills by self-employees in the media sector can be attributed to their communicative working environment. Their use of language suggests that stigma is a means to an end and most effectively deployed when hidden from or unrecognized by recipients. This is consistent with the research by Link and Phelan (3), who draw on Bourdieu to conceptualize stigma power processes. The results can be seen as a small contribution to the research gap mentioned by Link and Garcías (25) concerning advantaged groups, as the self-employees with high income are, and their role in creating health inequalities.

### 5.4 Scientific and practical implications

In our study, we contribute to the existing body of research by describing how different life worlds matter in shaping people’s conceptions of mental illness and stigmatization. Addison et al. point out that “stigma should be considered a social determinant of health because social relations structure an invisible reality” (49).

In a broader sense, we conclude that stigma is interrelated with societal processes (50). Previously, Yang et al. described the context-specific dynamics of stigma (5–7). Combining cultural contexts and values has helped to gain insight into stigmatizing attitudes toward people with mental illness (14). Based on our findings, we hypothesize that life worlds not only interact with stigmatizing attitudes, but also shape the stigma-process itself, which makes it difficult to measure and to compare. Our case study calls for research on stigma power and collective representations of mental illness in different life worlds.

Practical implications include the emerging interest in the interactions between life worlds and therapy. Kleineberg-Massuthe et al. (51) point out that social milieus are associated with symptom severity, treatment access and outcomes. Furthermore, Insel (52, 53) emphasized addressing the mental healthcare crises by focusing on the purpose of recovery. Finding a purpose seems more likely when taking people’s life worlds into consideration, and suggests the importance of purposes which are not driven by individualism, the social pressure to compete and the inclination to self-optimize.

A large part of the stigma research is based on questionnaires and quantitative data analyses. Similarly to Stutterheim and Ratcliffe (13), we recommend future qualitative research to better understand stigma’s complex outcomes, circumstances and related behaviors. Thinking further, language matters for both qualitative and quantitative data collection. We found that different language was used by people in different life worlds. Our results reveal that certain life worlds may be more difficult for researchers to access. For example, researchers may neglect the language used by people in different life worlds when developing questionnaires (including not only simple language). Researchers should different concepts of mental illness in different life worlds into account, for instance by including life world related examples for case vignettes. There is a particular need to establish sensitive measurements for facets of liberal values that enable stigmatization, but which currently cannot be reliably measured on a population level (54).

### 5.5 Strengths and limitations

Despite the small sample size and the selective groups, the analyses provide helpful insights into the development and manifestation of stigma toward people with mental illness in two different life worlds. Within focus groups, people were characterized by shared experiences and similar life goals. They shared a common language, enabling them to communicate with each other on an equal footing. This was facilitated by the fact that study participants in each group already knew each other. The small group size enabled study participants to contribute to the discussion. With our two contrasting focus groups, each with at least four participants, we fulfill the criteria for group sizes described by Krueger and Casey (26). Since we organized the focus groups as homogeneous groups based on participants’ life world—meaning all participants share the same life world—we assume the scope of perspectives captured is only partially influenced by the small group size, though this cannot be entirely ruled out. We found no evidence suggesting that the different durations of focus groups impacted the study results. Although our team of researchers discussed and reached a consensus on theoretical saturation, we would like to point out that there are no universally applicable guidelines for determining when saturation has been reached. Theoretical saturation is based on assumptions that we clarified in the section about participant selection. Furthermore, our semi-structured interview guide enabled study participants to add relevant topics that were not considered during the development of the project and helped them emphasize their most significant perspectives and thoughts. For the results section, the research team consented on which quotations to include to illustrate our interpretation of the dataset, as well as our codes and themes. However, these quotations represent only a small part of the focus group discussions.

The concession to (moral-) economic aspects of life worlds (mirrored in the large income differences between groups, for instance) as framework of the article does not limit its informative value in principle. However, it does reduce the presentation of everyday life of the interviewees.

Although though there was a fair agreement between the two coders (33), greater consistency would be possible. How we dealt with this methodological limitation is described in the section about the Coding Tree (section 4.2.). The discrepancies in the coding can attributed, in part, to the exploratory nature of the study and its open-ended approach. This is also evident in the large proportion of inductive codes. Unlike the defined deductive codes in the code book, these codes were more like impulses, which may occasionally caused uncertainty between the two coders. This is also related to the fact that the assignment of statements to higher or lower-level codes, as well as the length of the statements, may have differed between the two coders. To keep our analyses as complex as necessary and as simple as possible, we focused on maximum contrasts between two groups. Due to time constraints, the schizophrenia vignette was only briefly discussed in the group of self-employees and not discussed at all by the volunteer fire fighters. A native English-speaking colleague (AL) translated the quotations from the German transcripts, using the audio files for proofreading. However, we cannot rule out the possibility that this might slightly weaken the rigor of the qualitative data report. The original quotes, as well as the translations, can be found in Supplementary Table S1.

To operationalize life worlds, we relied on the results by Yang et al. (5), who studied Chinese immigrants in New York. The researcher state that one’s job and money matter most in moral approaches and stigma toward people with mental illness. However, groups based on occupational similarities and life goals are just one possible solution to operationalize life worlds or social structures. Other factors may also influence values and behaviors as well, such as mental health literacy and health behavior (55). These factors could serve as an interpretative lens for future studies. Here, we focus on the economization of the social sphere to understand stigmatization toward people with mental illness in different life worlds. For example, the participating volunteer fire fighters lived in rural areas in the East of Germany, and the self-employed participants lived in a major city in the North of Germany. Both groups referred to examples of regional differences, which may be interrelated with job opportunities as well as study participants’ professions. However, the focus groups did not discuss regional characteristics, so they are not part of our analyses. When comparing results with those of Otte (56), it is worth questioning whether and how life worlds are consequence of self-selection. More precisely, we could not answer how and if life worlds shape attitudes or how and if attitudes shape life worlds. As Lindner (57) states: “Just as the habitus makes the habitat, the habitat in turn reinforces the habitus, for example by attracting relatives of choice” (p. 511).

In sum, the sample size is small, but our case study provides first insights into stigmatization behavior in various life worlds. Many more life worlds need to be studied. While focus groups were a useful method to examine stigmatizing attitudes and behaviors, a discourse-analytical approach might be suitable for future research to investigate the interrelation of economization of the social sphere, society and stigma.

## 6 Conclusion

Our case study highlights the importance of the compiled theories and research on the economization of the social sphere in different life worlds and its influence on how people talk about mental illness and stigma. We observed that diverging modern societies can be researched as life worlds. These life worlds conceptualize more than socioeconomic status, age or sex as common descriptive categories for example. Future research should interrogate these issues more fully and clinicians might usefully consider people’s symptoms in the life worlds in which they emerge and are responded to.

Future surveys should generate items which take manifold life worlds, interaction spaces, everyday environments according to Schütz and Luckmann (9) and Zifonun (8), needs, goals, and related conceptions of mental illness into consideration as well as thoughts regarding discriminatory behavior toward people with mental illness. Survey methods should be aware of and investigate the economization of the social sphere to examine modern stigma processes and their implications for public health. Life worlds can be used to develop targeted education and intervention strategies to destigmatize mental illness, e.g., with the photovoice-projects (58). The interactions between life worlds and therapy should be researched and considered in healthcare.

## Data Availability

The raw data supporting the conclusions of this article will be made available by the authors, without undue reservation.
