# Supplementary material for: Exploring the stigma of mental illness in life worlds of different economic pressure: a maximal contrast analysis
Source: Front Public Health. 2026 May 18;14:1677166. doi: 10.3389/fpubh.2026.1677166 (PMC13223182; doi:10.3389/fpubh.2026.1677166)
Supplement: Supplementary file 1 [file Supplementary_File_1.docx]

Supplementary Material

**Supplementary Table 1**: German and to English translated illustrating quotations

|  | German Quotation | English Translation |
| --- | --- | --- |
| *1* | Na ja, das Wichtigste, was eigentlich oftmals nicht so stattfindet, wie man es sich wünscht, ist eigentlich die Ursachenforschung. Wir haben, mal so gesagt, wenn mir mein Mitmensch dann so über den Weg läuft in der Veränderung, dann sage ich mal, geht es ja meistens so, dass man immer so schön symptomatisch behandelt. So: Bist du niedergeschlagen, musst du Stimmungsaufheller nehmen. Mal gesponnen jetzt, irgendein Medikament oder so. Aber eigentlich ist es ja wichtig, mal in das Gespräch zu kommen mit den Leuten.  (D/4, Abschnitt 105) | Well, the most important thing, that doesn’t happen the way you want it to often, is actually looking for the root cause. Let me put it this way: when I come across another person who’s changed, then you usually really only treat the symptoms. Like: you’re in a bad mood; you have to take mood enhancers. Like, some medication or something. But what’s actually important is to talk to people.  (D/4, section 105) |
| *2* | Ja, die Menschen mit Behinderung sind für mich die ehrlichsten und dankbarsten. Und da kann man manchmal auch die Anforderungen ein ganzes Stück zurückschrauben.  (D/4, Abschnitt 30) | Yes, people with disabilities are the most honest and grateful people for me. And sometimes you can lower your expectations quite a bit.  (D/4, section 30) |
| *3* | wir sind eine Gemeinschaft. Wir wollen zusammen in das Feuer reingehen. Wir wollen zusammen wieder rauskommen.  (D/1, Abschnitt 61) | We are a community. We want to go into the fire together. We want to come out together.  (D/1, section 61) |
| *4* | Ich sage mal, da ist der Vorteil in der Regel, du hast da – wie ich immer so schön sage – in einer Blaulichtfamilie eigentlich ein gutes Netzwerk. Wir hatten einen derben Wohnungsbrand vor ein paar Jahren hier gehabt in [Ort, Erzgebirge], wo unsere Ortsfeuerwehr dann auch die Bergung von der Leiche gemacht hatte. Und der eine Kamerad, der hätte danach tüchtig zu kämpfen gehabt. Und von ihm ein Arbeitskollege, in einer anderen Feuerwehr tätig, der rief mich dann an und sagte: Mensch, ich weiß, ihr habt jetzt auch Weihnachtsfeier und alles. Tut noch einmal mit ihm reden, er gefällt uns gar nicht. So, und wo du dann die Chance hast, wenn du diesen Hinweis hast, dem auch nachzugehen und anzubieten und zu gucken, was kannst du selbst machen. Da gehört eben auch eine Flasche Bier abends dazu unter Männern oder auch Frauen.  (D4, Abschnitt 110) | I would say the advantage you usually have - as I always say - a good network in a “blue light family”. We had a serious house fire here in [town, Erzgebirge] a few years ago, where our local fire brigade also rescued a dead body. And one of our comrades struggled on this afterwards. And a colleague, who worked in another fire brigade, called me and said: "I know you are at the Christmas party. Please, talk to him again; we are concerned about his condition. So, if you have the chance, to talk to him or offering him, to talk to him. That also includes having a beer in the evening.  (D/4, section 110) |
| *5* | Befragter 1: Auf der anderen Seite glaube ich aber auch, dass unser Lebensstil dazu führt, dass immer mehr Leute krank werden. Weißt du, das ist so: Ja, wir leben irgendwie in einer Welt, wo man sich alles leisten kann und wo jeder unabhängig seinen Traum verwirklichen kann. Aber auf der anderen Seite glaube ich, dass das viele Leute immer unzufriedener macht, dieses: Ich bin in keiner Gemeinschaft mehr drinnen und ...  Befragter 3: Aber die Frage ist ja: Ist das klinisch diagnostiziert?  (E/1 & E/3, Abschnitt 496-497) | E1: On the one hand, I believe our lifestyle causes more and more people to get sick. You know, it’s like we all live in a world where you can afford everything and where everyone can independently pursue their dream. But on the other hand, I think that a lot of people are unsatisfied with the sense of „I’m not in a community anymore and…“  E3: But the question is: is that a clinical diagnosis?  (E/1 & E/3, section 496-497) |
| *6* | Befragter 3: Wie viel Aufwand möchtest du aufwenden, um in diese Welt einzudringen und zusagen: Will ich da was verändern oder nicht? Die Frage ist: Kannst du überhaupt was verändern?  (E/3, Abschnitt 455) | E3: How much effort do you want to put into entering (t)his world and saying: Do I want to change something or not? The question is: Can you change anything at all?  (E/3, section 455) |
| *7* | Es bleibt auch einfach eine Denkübung, solange man irgendwie nicht entsprechend handelt.  (E/2, Abschnitt 583) | it simply remains a mental exercise as long as you don't act accordingly.  (E/2, section 583) |
| *8* | Jeder hat ja irgendwo ein bisschen was. Aber die Frage ist tatsächlich, wenn du sagst Einschränkung: Ist das was, was man wirklich merklich wahrnimmt, wo du sagst: Wow, diese Person kann nicht, was wir jetzt vielleicht an 100 Prozent Kapazität funktionieren?  (E/3, Abschnitt 48) | Everyone has a little bit of something. But the question really is, when you say “limitation”: Is that something that you really, actually notice, where you say: Wow, this person can't function at 100 per cent capacity?  (E/3, section 48) |
| *9* | Befragter 2: Na ja, auch mit einem Depressionsszenario es manchmal am schwersten ist, denjenigen zu helfen, die nah an einem dran sind >  Befragter 4: Klar, weil man ... Sozusagen die Scham ist am größten von denen.  Befragter 2: Die Scham. Aber man möchte ihn ja auch irgendwie ... Man möchte ihnen ja auch irgendwie nichts antun, was sie nicht wollen, vermeintlich.  Befragter 3: Man sieht es vielleicht auch irgendwann gar nicht.  Befragter 2: Man sieht es vielleicht gar nicht. ()  (E/2-4, Abschnitte 324-328) | E2: Well, even with depression, it’s sometimes hardest to help those who are close to you >  E4: Sure, because you … The shame, so to speak, is the greatest of all.  E2: The shame. But you also want to somehow ... Somehow you don't want to do anything to them that they don't want, supposedly.  E3: You might not even see it at some point.  E2: Maybe you don't see it at all.  (E/2-4, section 324-328) |
| *10* | Befragter 3: Bist du derjenige, der sich kümmert, oder jemand anders?  (kurze Unterbrechung – )  Befragter 1: Sind wir da jetzt für verantwortlich, uns um ihn zu kümmern, und kriegen das auch irgendwie hin? Aber ist das Problem nicht auch so ein bisschen unsere Gesellschaft, dass wir irgendwie immer davon ausgehen, jeder ...  Befragter 3: Irgendwer wird es schon richten, meinst du? ()  Befragter 2: Es wird schon eine Ebene geben, die organisiert.  Befragter 1: Nein, dass man sagt, so Privatsphäre und ich muss dem sein ... Also wann überschreite ich seine Privatsphäre? Weil bei dem Beispiel jetzt einfach da anzurufen und zu sagen: „Hier, bitte einweisen“, ist ja auch ein krasser Ein... also ... Ähm ...  Befragter 2: Ja.  Befragter 1: Das ist dann auch immer so: Bevor ich was falsch mache, mache ich vielleicht einfach gar nichts >  (E/1-3, Abschnitte 343-349) | E3: Are you the one who takes care of him, or someone else?  (short pause)  E1: Are we responsible now for taking care of him, can we manage it? But is the problem not also a little bit our society, that we always assume that everyone…  E3: Someone will deal with it, you mean?  E2: There’ll be something that organizes it.  E1: No that you say, well, privacy, and I need to… like when do I invade his privacy? Like in the example [vignette], just calling and saying, „here, please get him to the hospital“, it’s just a blatant… well… um…  E2: Yeah.  E1: Then it’s always like: before I do something wrong I might as well do nothing at all.  (E/1-3, sections 343-349) |
| *11* | Ich habe immer so ein Problem mit Leuten, die nicht logisch denken, und wenn man so krank ist, dann denkt man halt eben nicht mehr logisch.  (E/1, Abschnitt 447) | I always have a problem with people who don't think logically, and when you're that ill, you just don't think logically anymore.  (E/1, section 447) |
| *12* | Im ersten Teil hatten wir ja schon dieses Sich-nicht-mehr-auf-Menschen-verlassen-Können, hat in einem beruflichen Kontext viel mehr Konsequenzen, würde viel mehr Konsequenzen haben. Also das, was [Name Befragter 3] eben gerade erzählt hat von seinem Kollegen, den er irgendwie auf den Job mitgenommen hat, der dann nicht geliefert hat und dann entsprechend raus war, das wäre bei mir genauso. Also wenn ich jemanden habe, der sagt irgendwie: Ich brauche das und das. Oder ich sage ihm: Ich brauche das und das bis übermorgen. Und es kommt zweimal, dreimal nicht und dann hat das auf jeden Fall zur Folge, dass ich mit dem wahrscheinlich nicht mehr zusammenarbeiten werde. Und wenn es jetzt zum Beispiel um meine Frau geht, da gilt es auf jeden Fall nicht, für die bin ich da, no matter what. So, und auch No-matter-what ist auch so ein bisschen die Frage, weil, irgendwann ist dann auch Schluss.  (E/2, Abschnitt 215) | In the first bit we already talked about not being able to rely on people anymore, which in a professional context has way more consequences. So that, what [E3] just said about his colleague that he took with him to the job, who didn’t deliver and then was out [of the job], it would be the same with me. Like, if I had someone who said: I need this and that. Or I tell him: I need this and that by the day after tomorrow. And if that doesn’t happen two or three times then it’ll definitely have the consequence that I wouldn’t work with that person anymore. And if that was, for example, my wife, then that wouldn’t be the case because I’m there for her no matter what. Well, no-matter-what is also kind of in the air because at some point that’s over, too.  (E/2, section 215) |
| *13* | D4: Also man muss dann irgendetwas einfallen lassen, wie ich die trotzdem bei Laune halte, um auch mal zu sagen: Du bist jetzt passives Mitglied mit den und den Aufgaben oder wir finden Möglichkeiten in anderen Feuerwehrbereichen. Es gibt ja … ich sage mal bis hin zu musiktreibenden Zügen, die wir haben. Vielleicht gibt es eine musikalische Lösung, wo man denjenigen weiter integrieren kann und, und, und. Da ist, sage ich mal, dem Improvisieren, was wir in der Feuerwehr eigentlich gut können, keine Grenze gesetzt. Das sind so die wichtigsten Punkte, die mir so für heute Abend durch den Kopf gingen, als wir uns … als dann die Anfrage von Ihnen oder von dir kam. () Und ja, da gibt es sicherlich auch richtigerweise hier gesagt, jetzt kein richtig und kein falsch. Also es ist immer eine individuelle Lösung, wo wir jetzt kein Lehrbuch hat schreiben können, sondern vielleicht eher, sage ich mal, einen Ideenkatalog, was irgendwo so jemand schon einmal probiert hat und gemacht hat, um diese Inklusion dann auch in dem Bereich zu ermöglichen.  Interviewerin: Und was wurde schon gemacht oder was sind so Vorschläge? Auch gerne, wenn ihr damit Erfahrung gemacht habt oder so.  D1: Ja, also wie gesagt, wir haben uns ja auch schon einen Kopf gemacht bei uns, auch die Kameraden. Also er wird auch weiterhin mitgehen dürfen, egal wann und wie. Weil, er ist ja Feuer und Flamme und er wird, wie der [Name D4] schon sagt, auch für rückwärtige Dienste mit eingesetzt. Ich sage mal, wenn wir ausrücken, soll er mit Küche machen oder so etwas.  (D/4, Interviewerin, D/1 Abschnitte 23-25) | D4: So you have to come up with something to keep them happy anyway, like saying: You're now a passive member with these tasks or we'll find opportunities in other areas of the fire service. There are ... I mean, we even have musical platoons. Maybe there's a musical solution where you can integrate someone further and so on, and so son. I'd say there's no limit to improvisation, which we're actually good at in the fire service. These are the most important points that went through my mind this evening when we ... when the enquiry came from you. And yes, there's certainly no right or wrong way to put it. So it's always an individual solution, where we can't write a textbook, but perhaps rather, let's say a catalogue of ideas of what someone somewhere has already tried and done to make this inclusion possible in this area.  interviewer: And what have you already done or what are your suggestions? Also, if you have experience with it.  D1: Yes, well, as I said, we have thought about it, the other mates as well. So he will still be allowed to go with us [to the fire brigade operation], no matter when and how. Because he's on fire and, as [name D4] says, he can do other duties [which are not at the scene]. I say, when we are called out for an operation, he is supposed to do the kitchen or something.  (D/4, interviewer, D/1 section 23-25) |

**Supplementary Table 2**: Coding tree and number and proportion of coded segments for maximum contrasting groups (D/1-4: n=4 volunteer fire fighters, E/1-4: n=4 self-employed participants in the media sector) and overall focus groups (n=21)

| *Nr.* | Code | | Derivation of themes | maximal contrasting groups | | | | overall | |
| --- | --- | --- | --- | --- | --- | --- | --- | --- | --- |
|  |  |  |  | *D/1-4* | | *E/1-4* | |  |  |
|  |  |  |  | Number of coded segments | Proportion of coded segments within the focus group (%) | Number of coded segments | Proportion of coded segments within the focus group (%) | Number of coded segments | Proportion of coded segments over all focus groups (%) |
| *1* | - 1. | - Experiences and dealing with visible and non-visible disabilities | Deductive | 1 | 0.6 | 10 | 4.3 | 26 | 2.4 |
| *2* | - 1.1. | - Experiences and dealing with visible and non-visible disabilities with people with disabilities in the family | Deductive | 10 | 5.5 | 1 | 0.4 | 20 | 1.9 |
| *3* | - 1.2. | - Experiences and dealing with visible and non-visible disabilities with friends with disabilities | Deductive | 0 | 0 | 3 | 1.3 | 9 | 0.8 |
| *4* | - 1.3. | - Experiences and dealing with visible and non-visible disabilities with colleagues with disabilities | Deductive | 39 | 21.5 | 6 | 2.6 | 86 | 8.1 |
| *5* | - 1.4. | - Experiences and dealing with visible and non-visible disabilities with people in a wider social environment | Inductive | 3 | 1.7 | 7 | 3.0 | 39 | 3.7 |
| *6* | - 2. | - Thoughts, association and conception (towards people with/) of depression | Deductive | 7 | 3.9 | 4 | 1.7 | 23 | 2.2 |
| *7* | - 3. | - Course of depression | Inductive | 6 | 3.3 | 3 | 1.3 | 24 | 2.3 |
| *8* | - 4. | - Particularly relevant points / depression symptoms | Deductive | 0 | 0 | 2 | 0.9 | 17 | 1.6 |
| *9* | - 5. | - Experiences with and expectations towards people with depression | Deductive | 4 | 2.2 | 5 | 2.2 | 14 | 1.3 |
| *10* | - 5.1. | - Experiences with and expectations towards people with depression in the family | Deductive | 0 | 0 | 5 | 2.2 | 21 | 2.0 |
| *11* | - 5.2. | - Experiences with and expectations towards friends with depression | Deductive | 0 | 0 | 5 | 2.2 | 22 | 2.1 |
| *12* | - 5.3. | - Experiences with and expectations towards colleagues with depression | Deductive | 9 | 5.0 | 6 | 2.6 | 43 | 4.0 |
| *13* | - 5.4. | - Experiences with and expectations towards people in a wider social environment with depression | Inductive | 0 | 0 | 0 | 0 | 17 | 1.6 |
| *14* | - 6. | - Images of society for depression (supposedly shared norms towards people with depression, perceived public opinions towards people with depression) | Inductive | 3 | 1.7 | 13 | 5.7 | 38 | 3.6 |
| *15* | - 7. | - Thoughts, association and conception (towards people with/) of schizophrenia | Deductive | 0 | 0 | 14 | 6,1 | 32 | 3.0 |
| *16* | - 8. | - Course of schizophrenia | Inductive | 0 | 0 | 4 | 1.7 | 11 | 1.0 |
| *17* | - 9. | - Particularly relevant points / schizophrenia symptoms | Deductive | 0 | 0 | 5 | 2.2 | 13 | 1.2 |
| *18* | - 10. | - Experiences with and expectations towards people with schizophrenia | Deductive | 0 | 0 | 2 | 0.9 | 6 | 0.6 |
| *19* | - 10.1. | - Experiences with and expectations towards people with schizophrenia in the family | Deductive | 0 | 0 | 3 | 1.3 | 11 | 1.0 |
| *20* | - 10.2. | - Experiences with and expectations towards friends with schizophrenia | deductive | 0 | 0 | 4 | 1.7 | 8 | 0.8 |
| *21* | - 10.3. | - Experiences with and expectations towards colleagues with schizophrenia | Deductive | 0 | 0 | 1 | 0.4 | 12 | 1.1 |
| *22* | - 10.4. | - Experiences with and expectations towards people in a wider social environment with schizophrenia | inductive | 0 | 0 | 2 | 0.9 | 10 | 0.9 |
| *23* | - 11. | - Images of society for schizophrenia (supposedly shared norms towards people with schizophrenia, perceived public opinions towards people with schizophrenia) | Inductive | 0 | 0 | 4 | 1.7 | 7 | 0.7 |
| *24* | - 12. | - Conclusions: societal moral concepts of mental health / mental illness and behavior towards people with mental illness (e.g. as encroaching described helping behavior towards people with mental illness) | Inductive | 12 | 6,6 | 10 | 4,3 | 52 | 4.9 |
| *25* | - 13. | - Conclusions: personal moral concepts of mental health / mental illness and behavior towards people with mental illness (e.g. helping people with mental illness as reciprocal altruism) | Inductive | 13 | 7.2 | 10 | 4.3 | 57 | 5.4 |
| *26* | - 14. | - Conclusions: Cost-benefit analysis for helping people with mental illness (e.g. sharing responsibility health care system) | Inductive | 2 | 1.1 | 13 | 5.7 | 49 | 4.6 |
| *27* | - 15. | - Conclusions: Ranking of depression and schizophrenia, differences in dealing with people with depression and schizophrenia | Deductive | 0 | 0 | 10 | 4.3 | 25 | 2.4 |
| *28* | - 16. | - Conclusions: Similarities in dealing with people with depression and schizophrenia | Deductive | 0 | 0 | 8 | 3.5 | 9 | 0.8 |
| *29* | - 17. | - People with mental illness and freedom | Deductive | 2 | 1.1 | 4 | 1.7 | 14 | 1.3 |
| *30* | - 18. | - Characterization of focus groups by contents: description of living environment, as well as norms, values, life goals etc. | Inductive | 46 | 25.4 | 44 | 19.1 | 199 | 18.7 |
| *31* | - 19. | - Characterization of focus groups by language: semantic field, metaphors | Inductive | 24 | 13.3 | 22 | 9.6 | 149 | 14.0 |
|  | | | *Σ* | *181* | *100.0* | *230* | *100.0* | *1063* | *100.0* |
|  |  |  | *Proportion of non-coded segments (%)* |  | *29.0* |  | *21.0* |  | *30.0* |
